# Participatory co-creation of an adapted physical activity program for adults with moderate-to-severe traumatic brain injury

**DOI:** 10.3389/fresc.2022.900178

**Published:** 2022-08-04

**Authors:** Enrico Quilico, Shawn Wilkinson, Lindsay Duncan, Shane Sweet, Evelyne Bédard, Eric Trudel, Angela Colantonio, Bonnie Swaine

**Affiliations:** ^1^ Rehabilitation Sciences Institute, Faculty of Medicine, University of Toronto, Toronto, ON, Canada; ^2^ Centre for Interdisciplinary Research in Rehabilitation of Greater Montreal (CRIR), Montreal, QC, Canada; ^3^ Applied Human Sciences, Concordia University, Montreal, QC, Canada; ^4^ Kinesiology and Physical Education, McGill University, Montreal, QC, Canada; ^5^ Les YMCA du Québec, Montreal, QC, Canada; ^6^ Dalla Lana School of Public Health, University of Toronto, Toronto, ON, Canada; ^7^ The KITE Research Institute, Toronto Rehabilitation Institute, University Health Network, Toronto, ON, Canada; ^8^ École de réadaptation, Faculté de Médecine, Université de Montréal, Montreal, ON, Canada

**Keywords:** traumatic brain injury, TBI, physical activity, PA, program, community, participation, case study

## Abstract

**Background:**

Research about using physical activity (PA) to improve health, quality of life, and participation after moderate-to-severe traumatic brain injury (TBI) is receiving growing attention. However, best-practices for maintaining PA participation after TBI have yet to be defined. In this context, a team of researchers and stakeholders with a moderate-to-severe TBI (including program participants and peer mentors) participated in a co-creation process to optimize a 9-month, 3-phased, community-based, adapted PA program named TBI-Health.

**Purpose:**

The study aimed to provide a detailed account of the participation in and co-creation of a new TBI-Health Program to enhance sport and exercise participation for adults with moderate-to-severe TBI. Specifically, we carried out an in-depth exploration of the perceived experiences and outcomes of users over one cycle of the program to assist the co-creation process.

**Methods:**

An interpretive case study approach was used to explore the experiences and outcomes of the participatory co-creation within and across phases of the TBI-Health program. A purposeful sample of fourteen adults with moderate-to-severe TBI (program participants *n* = 10; peer mentors *n* = 4) were involved in audio-recorded focus groups after each program phase. Reflexive thematic analyses within and across the phases identified three higher-order themes.

**Results:**

Program Participation included barriers, facilitators, sources of motivation and suggested modifications to optimize the program; Biopsychosocial Changes highlighted perceived physical, psychological, and social outcomes, by self and others, that resulted from program participation; PA Autonomy emphasized transitions in knowledge, sex- and gender-related beliefs, and abilities related to exercise and sport participation.

**Conclusions:**

Study findings suggest the TBI-Health program can increase autonomy for and reduce barriers to PA for adults with moderate-to-severe TBI, which results in increased PA participation and important physical, psychological, and social benefits. More research is needed about the TBI-Health program with larger samples.

## Introduction

Research about using physical activity (PA) to improve long-term problems (e.g., cognitive impairment, depression, and quality of life) after moderate-to-severe traumatic brain injury (TBI) has received growing attention (1–3) and suggests that various forms of community-based PA may lead to significant health improvements (4). In addition, based on the long-term or lifelong injury related sequelae that can extend for 10–20 years after moderate-to-severe TBI, PA is particularly relevant as a self-management tool in the chronic period of recovery (5), as it has been shown that adults with moderate-to-severe TBI can, with minimal guidance, perform vigorous community-based PA (6). Although best-practices for maintaining community-based PA after moderate-to-severe TBI have yet to be defined (7, 8), participatory approaches to developing PA programs (e.g., partnering with stakeholders) are more successful because they are tailored to the unique needs and resources of the target community (9). Participatory approaches to developing health-related programs after TBI can also lead to additional outcomes based on the collaboration itself, such as increased cultural competency (appropriate communication with people of other cultures), community capacity (social capital), and ownership of the program (10). In this way, problem-solving with key stakeholders promotes their participation because they become active agents in the process.

Taking a participatory approach to modifying a PA- and nutrition-based program for the TBI community has been documented in previous research. For instance, consulting with an advisory committee of key stakeholders helped specifically tailor the content and delivery of an evidence-based diabetes prevention program for adults with moderate-to-severe TBI (11). A 10-member advisory committee, consisting of people with TBI, caregivers, TBI professionals, and program developers was provided with the curriculum of the original program two weeks prior to meeting. Then, during a structured, full-day consultation, qualitative information about modifying the content, format, and delivery of the program was collected to provide the necessary adaptations. More specifically, key features of that evidence-based diabetes prevention program were modified to include appropriate caregiver involvement, TBI-specific PA, nutrition recommendations, as well as content and eligibility criteria. When the program was implemented and evaluated, significant health-related improvements were found (e.g., decreased blood pressure, waist circumference), in addition to an 82% program retention rate over 12-months (12), which demonstrates how consulting with stakeholders can optimize both program outcomes and participation for adults with moderate-to-severe TBI.

In a similar participatory context, a team of researchers, community stakeholders, and persons with moderate-to-severe TBI (including program participants and peer mentors) collaborated for the purpose of optimizing a 9-month, 3-phased, community-based PA program named TBI-Health. The grass-roots initiative was first established in response to a need for PA support in the community after moderate-to-severe TBI (13) and was piloted from 2017 to 2019 to promote exercise and sport participation for its users. An initial exploration of the program revealed its potential, based on perceptions of the program's accessibility, benefits, and positive outcomes (14). However, a subsequent need was identified to cocreate a formal version of the program that could be replicated and evaluated for a larger cohort of users. Therefore, the purpose of this qualitative study was to provide a detailed account of the experiences and outcomes of the participation in and co-creation of a new version of the *TBI-Health* program to enhance sport and exercise participation for adults with moderate-to-severe TBI. Specifically, we carried out an in-depth exploration of the perceived experiences and outcomes of program users and peer mentors over one 9-month cycle of the program and co-creation process.

## Materials and methods

### TBI-Health program

**Figure 1:**
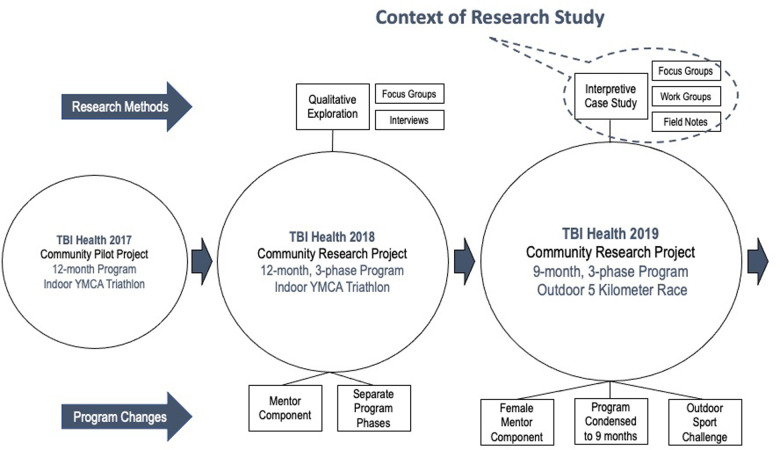
Context of research study.

The third iteration of the TBI-Health program discussed here (See Figure 1) was first developed by the Principal Investigator (PI; male) in partnership with the YMCAs of Quebec, and Quebec TBI Association. Its aim was to provide an inclusive, community-based environment that facilitated exercise and sport participation through peer-assisted activities, group dynamics, and autonomy-supporting behaviors so that French- and English-speaking adults with TBI could obtain the benefits associated with PA outside the clinical setting. The 9-month TBI-Health program was led by the PI, ran every Tuesday and Thursday morning for 90 min, and progressed through three broad phases, focusing on how to exercise (1) *safely* in a smaller machine-based section of the gym, (2) *independently* in the larger free-weight and machine-based section of the gym, and (3) *for a sport challenge* on an outdoor walking trail near the gym.

Group-based exercises and instruction during TBI-Health were delivered by the PI, a certified personal trainer at the YMCA. In phase one (weeks 1–12), group programs took a generic approach to prescribing 10–15 min of warming up on cardiovascular machines, before completing 8–10 resistance training exercises (2–3 sets of 12–15 repetitions) on major muscle groups of the body for 45–60 min in a smaller gym located on the second floor of the YMCA Center. The sessions always finished with body-weight exercises for the core muscle groups and stretching to cool down in a studio for 20–30 min.

Phase two (weeks 13–24) followed the same approach but took place in a larger gym with more equipment, located on the third floor of the YMCA Center. Similar programs were provided to the participants through mobile fitness applications, which they accessed on their personal mobile devices. In phase three (weeks 25–36), participants continued their individual programs on the third floor in addition to following a progressive run/walk program to prepare for a community organized, 5-kilometer (k) sport challenge on an outdoor walking trail near the YMCA. Exercises across phases were modified for participants with mobility limitations from an ability perspective (e.g., participants with arm-based mobility limitations were encouraged to use leg-based exercise machines). Participants were also encouraged to exercise at the center outside program hours, but adherence was measured by program attendance. Motivational strategies were regularly provided in the form of positive encouragement by the PI and peer mentors (past participants from the program).

The starting level for cardiovascular and resistance training exercise was established by the participants, while supervised by the PI, based on their perceived rate of exertion. Subsequent progressions were guided by incremental increases in time and intensity of approximately 10% per week. There was no home program component, but non-exercise components included social activities organized between members and a social media page that facilitated discussions between past/present participants in the program. The PI kept a written log about participants' involvement, fidelity, and attendance during sessions, in addition to any adverse events.

### Study design

The study was guided by participatory and interpretive case study methods to provide a multi-faceted exploration of a particular case over time through an in-depth collection of multiple sources of evidence in a real-life context (15, 16). The design aligned with the PI's interpretive and participatory worldview, where scientific inquiry is conducted with consumers, who ultimately benefit from the participatory effort (17). Program participants and peer mentors with moderate-to-severe TBI were actively engaged in the knowledge generation and transfer process through participatory work groups and focus groups that tailored the program to support their own sport and exercise participation as it was being delivered. The interpretive paradigm allowed the research team to explore the participants' lived experiences through a relativist ontology (people create a mental construction of their worlds through lived experiences) and constructivist epistemology (knowledge is both subjective and constructed). Like participatory research that explored PA experiences of Aboriginal youth (9), our approach aimed to facilitate an exploration of the parallel experiences and outcomes of both participating in and co-creating the adapted PA program for adults with moderate-to-severe TBI. The COREQ checklist for reporting qualitative research aided the preparation of this manuscript (**Supplementary File S1**; 18).

### Participants

Through convenience and purposive sampling, 17 adult participants with moderate-to-severe TBI (10 male, 7 female) were recruited to participate and contribute to the new *TBI-Health* program. Eligible participants (1) were at least 18 years of age, (2) lived with a moderate-to-severe TBI, (3) and were able to speak English or French. Exclusion criteria were (1) not being able to safely participate in general forms of PA, (2) having a heart, blood, or respiratory conditioning that could be worsened through PA, and (3) not being an in-or out-patient in a rehabilitation hospital center. Recruitment was facilitated by the Montreal-based Quebec TBI Association, based on their members’ medical records, which were kept on file. Program participants included six new (2 male, 4 female) and four returning members (4 male), in addition to four peer mentors (3 male, 1 female). One participant was partially hemiplegic, two participants used a walking cane, and another wore an ankle brace. All participants could communicate effectively and had sufficient cognitive ability to understand program instructions and travel independently to and from the YMCA. Peer mentors were past participants onboarded as YMCA volunteers to help run the program as expert members and positive role models for the others. Demographic characteristics of the 17 participants are included (See Table 1). Study participants’ names were coded to keep their identities confidential.

**Table 1:** Demographic information about study participants.

Participant	Sex	Age (yrs.)	Education	Marital status	TBI	TSI (yrs.)	Etiology
M1	Female	27	Secondary	Single	Moderate	2	MVA
M2	Male	50	University	Married	Severe	20	MVA
M3	Male	38	Secondary	Single	Severe	11	MVA
M4	Male	44	No diploma	Single	Severe	38	MVA
P1	Male	40	University	Case Law	Severe	7	Assault
P2	Female	59	Secondary	Divorced	Severe	10	MVA
P3	Female	19	Secondary	Single	Severe	4	MVA
P4	Male	36	College	Divorced	Severe	21	Fall
P5	Female	27	Secondary	Single	Severe	10	MVA
P6	Male	46	University	Single	Severe	2	Assault
P7	Male	48	College	Other	Severe	14	MVA
P8	Female	37	College	Case Law	Moderate	18	Fall
P9	Female	27	No diploma	Other	Severe	21	MVA
P10	Male	53	Secondary	Married	Moderate	2	MVA
P11	Male	36	No diploma	Single	Severe	21	MVA
P12	Female	33	University	Other	Severe	8	MVA
P13	Male	46	University	Single	Severe	21	MVA

M, Mentor; P, Participant; Yrs., Years; TSI, Time Since Injury; MVA, Motor Vehicle Accident.

### Data collection

**Figure 2:**
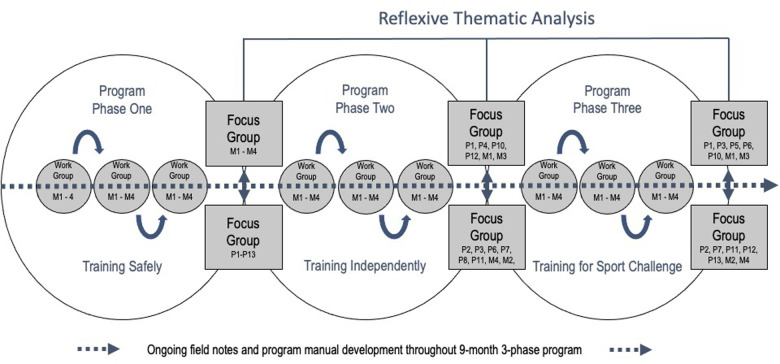
Participatory cocreation process.

The process of co-creation (involving capturing participant experiences) was conducted over 9-months of program delivery through participatory work groups, focus groups, and field note observations. Whereas the nine monthly participatory workgroups tailored the content and delivery of each phase as it was being delivered, the focus groups at the end of each 3-month phase reflected the perceived experiences of all the participants involved (See Figure 2).

### Participatory work groups with peer mentors

In addition to their leadership roles with the program, the four peer mentors collaborated with the research team through nine work groups, lasting approximately 60 min each, in a private office at the YMCA with the PI and a senior member of the research team (BS/SW). Work groups were structured with five guiding questions about how (1) the program was running, (2) the group was responding, (3) program components should be modified, (4) the mentors’ responsibilities should be defined, and (5) they would self-rate their contribution to the development of the program in each meeting on a scale from 1 to 10 (10 being optimal and 1 being minimal contribution to the session). Individual responses to the questions in the work groups were recorded in written text, with a pen, on paperback journals assigned to each one of the mentors. Data from these sessions were not analyzed, but rather integrated within the modules and delivery of the program as it was being offered (e.g., focus of each program phase, reminders about program exercises, incorporating team building activities outside program hours).

### Focus group interviews

Program participants and peer mentors were involved in audio-recorded 60–90-minute semi-structured focus groups at three time points across the 9-month program, approximately three months apart, after each respective phase of the program. Focus groups took place in a private studio at the YMCA and were divided in two sections to accommodate the number of people and provide the opportunity to discuss individual and shared experiences (6 total). They were led by the PI, who had previous experience interviewing adults with moderate-to-severe TBI (13), and a senior member of the team (SW). Focus groups were first divided by mentors and participants to obtain insights about their similar/different experiences. After the first data collection time point, however, participants and mentors asked for the groups to be combined, as they believed it provided a more accurate representation of their shared experiences.

The semi-structured question guide and probes for the focus groups were based on a question guide developed with the multidisciplinary team's expertise and piloted with past participants for optimal length and prompts. The questions focused on gathering perceptions about (a) experiences, including program impacts and sex and gender considerations; (b) positive/negative aspects of each phase of the program, including what was successful, challenging, and required modification; and (c) any personal changes that had occurred in that phase, in relation to their capabilities, opportunities, and motivations. Interview questions across the three time points were the same to achieve conceptual depth about the peer mentors and program participants’ experiences and outcomes in the participatory co-creation process.

### Field note observations

The PI supervised the TBI-Health program two days per week over a period of nine months for a total of 72 of the 75 program sessions (∼108 h). He was involved as a program coordinator to onboard, guide, and monitor the participants throughout the different phases of the program, but also maintained the role of participant observer throughout the 90-minute program sessions. As an observer, he walked around and spoke with participants to note his perceptions of their behaviors and gain entry to their everyday program experiences. During and after program sessions, the PI typed these observation and field note entries in a personal journal on his laptop computer, along with preliminary interpretations about his perceptions. The PI maintained similar field notes before and after each one of the participatory work groups and audio-recorded focus groups, to document contextual information about the meetings and his possible influence on participants in his dual role as PI and program coordinator.

### Data analysis

Braun and Clarke's (19) reflexive thematic analysis of the focus group data allowed us to identify, analyze, and report patterns (themes) within participants’ experiences of both co-creating and participating in the program within and then across the three phases of the program. Although grounded theory approaches are traditionally associated with participatory research, we determined that an inductive reflexive thematic analysis was more appropriate because our research questions did not center on social processes, nor did we intend to develop a grounded theory from the data, or sample theoretically (20). The audio-recordings of the focus groups were transcribed verbatim by a professional transcriptionist (female) and the analyses were carried out through an iterative process between a female RA with graduate degree in neuroscience, the PI, and four co-investigators (Co-Is; 2 male, 2 female) on the research team, who were all bilingual.

To capture the perceived changes about the participants’ perceptions over time, the analysis team determined that pooling the data and systematically coding by phase of the program would facilitate the interpretive goal of generating intra-analysis themes *within* phases, before developing inter-analysis themes *across* phases. The *first step* of this analysis involved becoming familiar with the data by reading and re-reading transcripts, while taking notes about initial ideas. In the *second step*, a codebook was generated using the qualitative analysis software (NVivo 12). Once all segments were coded, the RA met with the PI to carefully discuss and revise the codes, and ensure they accurately represented the semantic content. In *third step*, the RA began collating the codes into potential sub-themes within each phase, while maintaining consistent language to help identify similarities and differences across cases.

For the *fourth step*, the RA, PI, and Co-Is worked closely through an iterative process of critically reviewing the sub-themes to ensure they accurately represented the coded extracts within each phase and the larger data set, in addition to discussing the direction of analysis, and guiding research question. The *fifth step* involved refining the similarities and differences of sub-themes, within and then across the phases, which revealed the types of changes that were occurring over time. Sub-themes identified from the intra-analyses within each phase were reviewed and defined before themes were identified from the inter-analyses across phases.

The final *sixth step* involved producing a graphic report of the intra- and inter-analysis process (**Supplementary File S2**), as well as compiling vivid and compelling extracts relating back to the research question. In this final step, the analysis team critically reviewed every step of the process, ensuring that each intra-phase sub-theme did not belong to a different inter-phase theme, by asking whether the sub-theme itself was problematic or needed to be re-named. The analysis was finalized by reviewing the final themes in relation to the data set and the research question to ensure that the overall story of analysis was reflected within the report. Included quotes were translated by a professional translator (female), with no prior connection to the participants.

### Trustworthiness

Ongoing methods were upheld to ensure the qualitative rigor of this study (21, 22), such as (1) prolonged engagement and persistent observation in the field; (2) peer-debriefing and reflexivity throughout data collection and analysis; as well as (3) data and analyst triangulation. The PI engaged with the participants as a program coordinator in 96% of the sessions across the 9-month period and reported about their behaviors through his field note entries during and after each session. This permitted regular observation of the participants and further developed his rapport with the people involved. Peer debriefing with Co-Is occurred before and after work and focus groups to critically assess the rigor in each step and enhance the PI's reflexivity about his dual role as researcher and coordinator. Reflexive logs allowed the PI to identify concerns about possible influences he may have had on the participants (e.g., positivity bias) and subsequently mitigate those concerns by consulting with senior members of the research team. For data triangulation, we presented quotes across peer mentors and program participants, to demonstrate the consistency of our findings across multiple sources. For analyst triangulation, the four Co-Is acted as the RA and PI's critical friends to help ensure the findings were transparent and coherent in every step of the process, which enhanced the credibility of our findings.

## Results

All four peer mentors were present during the nine participatory work groups and their self-rated contributions to each session ranged from 1 to 9.3 (M = 6.99; SD = 1.94). Program attendance varied per participant over the 9-months and 75 sessions (M = 46.64; SD = 16.89). Three participants (P8–9, P4) stopped attending for personal reasons after three, four, and six months of the program respectively, but the 14 others were involved in the 9-month project until the end. No adverse (undesirable) events were reported throughout the program. Eleven individuals (79%) participated in the 5k sport challenge, indicating the success with which the program was carried out as planned. Stemming from the six, semi-structured focus groups, three higher-order themes (***Program Participation, Biopsychosocial Changes,* and *PA Autonomy****)* were generated from the inter-analyses across the program phases, representing 10 sub-themes developed from the intra-analyses within each program phase, and 123 initial codes identified from the exploration (See Table 2).

**Table 2:** Themes and sub-themes with number of codes.

Program participation	Biopsychosocial changes	Physical activity autonomy
Program Barriers (8)	Cognitive Outcomes (2)	Physical Activity Capability (10)
Program Facilitators (6)	Physical Outcomes (14)	Sex and Gender Considerations (7)
Program Motivation (19)	Psychological Outcomes (16)	
Program Modifications (23)	Social Outcomes (18)	

### Program Participation—“It’s motivating to see people like us with traumatic brain injuries who are empowered to do more” (P12)

The ***Program Participation*** theme revealed the barriers, facilitators, sources of motivation, and suggested modifications related to optimizing program success and PA participation. Throughout the 9-month program, participants discussed the internal and external factors that affected program accessibility, experiences, and attendance, as captured in the *Program Barriers* sub-theme. In phase one, internal factors that related to program accessibility ranged from scheduling conflicts to communication difficulties due to second language proficiency and experiencing anxiety about participating in the PA correctly. P12 explained, “sometimes you look at those (members) there with big muscles, and a little voice inside you says that’s putting the bar high.” Similarly, in phase two, M3 explained how “it’s not written in our forehead that we have a TBI, you know. You can only see it because sometimes we have difficulties, or we get tired very fast – chronic fatigue.” Communication difficulties were mentioned again in phase three, so M1 emphasized that “when there is an important message, maybe you can translate it to me because most of them (participants) speak French, but I don’t.”

In phase one, external barriers included difficulties getting to the YMCA (e.g., public transit, parking). P7, who lived in the suburbs, explained that “it’s getting here that’s complicated … As my mobility is more reduced than for the rest of you. It’s accessibility. Finding a parking place, then getting to the gym, for me is the greatest barrier.” Participants also experienced difficulties when the smaller gym area was overcrowded because they felt that space was limited. In contrast, the external barrier experienced by some participants in phase two related to fewer opportunities for social interaction. P12 shared, *“*I love the machines on the third floor, but it seems that the people there are just interested in their own thing, because they don’t talk, they’re … uh, concentrated.” Whereas M3 further explained that building connections (with other members) just required a little more time, “It will be three years that I’m on the third (floor), since the beginning of the program, and it took ages before I could engage in a conversation with those people.” No external barriers were identified in phase three.

Throughout the 9-month program, participants discussed the external factors that affect program participation, in addition to the structure (e.g., schedule, reminders, support) and technology aids that make the program easier to follow and affect program experiences, as highlighted in the *Program Facilitators* sub-theme. In phase one, the participants discussed external facilitators related to receiving support and guidance from the mentors, as well as the YMCA staff and research team. P13 explained how, *“*the major success of phase one was the structure, like say putting a frame around a picture, which was provided by you and the whole team to facilitate things for us.”

Participants appreciated the program aids in phase one, like having the training program written on a visible whiteboard. M4 remarked: “it just makes things easier and … well that’s it. And with all those little reminders about the major muscle groups, or the rules, things like that.” But in phase two, the external facilitators related more to the progression of the program and technology aids, which were both specific to the transition to the larger gym area and use of an online software to facilitate the use of digital programs on the participants' smart devices. M4 explained, “the visual aspect, and that it's well explained  …  each rhythm is explained, then each muscle it activates is shown … Then you can also see the training progress over time, which is also interesting.” P2 added that having access to a mobile training application “gave us more autonomy.” External facilitators were not identified in phase three.

The sources of motivation for participating in the program varied considerably and in relation to the changing focus of the phases in the *Program Motivation* sub-theme. Participants' motivation in phase one was related to biopsychosocial outcomes, the structure the program provided in the week, being an example for others with TBI, and contributing to the research project. For instance, P13 explained the way he felt: “participating in the research is truly motivating … the altruistic part of me and that part that wants to help the brain injured also comes in when considering the possibility of developing a post-rehabilitation reference that may be recognized and publicized.” Motivation in phase two related again to the perceived biopsychosocial outcomes and structure in the week, but was extended by accountability and an increased interest in PA. For example, P4 discussed how: “everyone is more motivated in phase two. In phase one, you knew it was good for you, so you went. But ahhh … by phase two, people are happy to be there!”

Participants also expressed how being around others from the group helped maintain their motivation to participate in PA. P10 shared, “if it’s me who is deciding on my own, it won’t work. I can’t get there. Someone must help me … push me to go forward. That encourages me (talking about the group).” P4 also shared, “Simply put, it’s the ripple effect. When we see people like us training, it pulls you into the wave.” In phase three, however, the motivation for participating in the program was more related to having a training routine, pushing personal limits, and the 5k sport challenge. P6 explained: “it motivates me more because it means the moment approaches when we leave the YMCA. The fact of going out to the mountain, with no machines. You run or you walk … you are with nature.” Similarly, M1 added: “I enjoy it because … it’s challenging. I never did that (a community sport challenge) before and then … you started to join with a group and meeting a lot of people.”

Suggestions from participants about improving the program structure across the three phases of the program are grouped in the *Program Modifications* sub-theme*.* A variety of recommendations for improving phase one of the program were provided, such as maintaining the program's structure while encouraging more freedom of discovery, including personal training goals and group activities, in addition to advertising the program externally, and clearly defining the mentors' roles and responsibilities. Confusion about the mentors' roles and responsibilities was discussed on several occasions, in addition to the mentors' desire for more responsibility. M4 explained, “I’d rather have a framework with precise objectives to respect, so I have a title and suitable role as a mentor*.*” On several occasions, mentors also expressed their desire to proudly represent the group through TBI-Health logos on their clothing, in addition to a desire to give back to the community. M2 shared, “it’s like we’ve become … we want people to become aware, for example, of the accident we’ve had, and everything to do with TBI.”

Further suggestions during the focus groups about improving program structure in phase two included maintaining the existing progression across phases, including progressions within phases, adding a nutritional component, scheduling group classes once a week, and adapting the walks outside the center by walking speed and ability. M3 shared, “I would love if it would be possible just to go up the mountain at your own speed, and to have a breakpoint … somewhere where we all meet together no matter what.” Suggestions about improving program structure in phase three included providing access to YMCA trainers and classes, in addition to maintaining the program's 9-month length and clearly defined phases. M3 offered his impression of why this approach works: “I feel that basically, the program is like a cooking recipe and basically if you carefully list how to add all the ingredients, well everyone will make a success of the recipe.”

### Biopsychosocial Changes – “When I walk, I have more confidence in myself because I am more stable. I lose my balance less” (P5)

The ***Biopsychosocial Changes*** theme reflected the perceived changes, by self and others, related to the physical, cognitive, psychological, and social outcomes from the program. The *Physical Outcomes* sub-theme in phase one reflected the participants' perceptions about their improved physical endurance and management of TBI impairments related to sleep, energy, and recovery as well as the perceptions of others. Some were not aware of these potential benefits. For instance, P2 explained: “I never thought … that physical activity would make me progress that well. I have so much more energy than before.” Perceptions about physical outcomes in phase two had more to do with strength and endurance, in addition to the management of TBI-related impairments with balance, mobility, and migraines. This was the first time that participants spoke about these kinds of changes, which they related specifically to the program. P8 shared, “the workout that you developed for us unblocked something in my back. So that now, I can walk straight. I no longer need a brace, no longer need a cane, so for me it’s just like, wow!” In phase three, perceptions about improvements in physical abilities were extended with cardio and endurance in addition to the management of TBI related impairments with balance, movement, mobility aids, and migraines. P5, who is partially hemiplegic explained “when I walk, I have more confidence in myself because I am more stable. I lose my balance less often … I like that better.” In addition, P3 no longer used a cane following the program, and P12 no longer required her orthotics (ankle brace).

In addition to physical outcomes, and beginning in the second phase, participants spoke about their perceived improvements with concentration and memory within the *Cognitive Outcomes* sub-theme. M1, explained that in phase two “I’ve started memorizing, I feel like my brain is working now. Before, like every second … I always checked in the paper that you gave me what’s next … But now, I know what I need to do once I get inside, I know what I need to do.” Similar examples were shared in phase three by P6:

We're not less intelligent than other people. Personally, I tend to forget things. It doesn't make me less capable of understanding. But the fact of training upstairs, more than on the second floor, gives us more opportunities to use our memory and remember the exercises.

M3 further emphasized, “the more important muscle is up there (the head). And that’s why we’re training. And that’s why we’re doing this study as well … I think the concentration and I think that the memory is working hard as well as we’re training.” Findings about cognitive outcomes, which surfaced in phases two and three, were not identified in phase one of the program.

Within the *Psychological Outcomes* sub-theme, participants expressed perceptions by self and others about positive changes to their mood states related to self-confidence, feelings of enjoyment, pride, and privilege, as well as feeling valued and validated as someone with a TBI in phase one*.* M3 shared his sentiments:

It makes me proud to take part as mentor in a research study about the subject that has been the most terrible experience of my life, the accident that nearly killed me … and to have saved myself and to have gotten where I am today and the people around me, my family, are so proud of that, and when I tell them about it, and how we participate, you know, we're here! I hold that close to my heart.

These positive feelings were mirrored by the impressions of others too, as seen with P2’s personal example: “I started the program in April, and my daughter, two or three weeks ago said to me: mom, you’ve changed so much. You’re so much better, she says. You’re starting to be more … she thinks I’m slowly becoming as happy as I was before.”

In phase two, the positive perceptions about the participants’ mood states were extended to feelings of capability, empowerment, motivation, physical improvements, and having a positive role in the program. Participants shared how they felt accomplished and empowered to carry out more daily activities, which would have otherwise been restricted by fatigue. They were energized and motivated about their perceived physical improvements and felt positive about helping others in the program. P4 said, “it brings me, well, a lot of pleasure, but it also brings me a satisfaction, like a feeling of duty accomplished.” Phase three included positive changes to mood states related to self-care, pride, and capability of completing the outdoor 5k sport challenge. P7 explained, “me personally, I felt surprised, I never thought I’d do so well …” The mentor M2 added, “as for the (sport) challenge, it was really awesome. Outdoing yourself … a great success, and why not do a bigger challenge (in the future)?”

The *Social Outcomes* sub-theme was identified the most frequently within the ***Biopsychosocial Changes*** theme. In the first phase of the program, participants discussed examples related to their program participation together, indicating an area that was relevant to their lives. They recalled experiencing positive social time with non-program participants, as well as with program-peers, in addition to feeling equal to other members at the YMCA. M3 explained, “People from the Y here, we’re quite different from them, as we’ve seen. They do not look down on us, nor do they look up to us. We’re equal with everyone, we have our place here … this is where I discovered it, in fact.” They also compared their tight-knit group to a family, due to the inclusive nature of the program. In phase one for instance, they discussed the self-improvements that resulted from their shared experiences together. M1, who was encouraged by her peers to become a mentor explained, “yes … I did improve a lot. Before like M2 said, I’m just always shy, hiding … I don’t talk to anyone that I don’t know, but now I’m starting to talk to someone (in reference to the young man she is in a relationship with).” In phase one, the mentors asked to join the participants in future focus groups. M4 explained, “I don’t feel my title makes me any better than the others. I’m … lucky enough to be a mentor because I’ve been here since the first cohort … but I don’t feel less or better than anyone else. I think we’re all on the same level*.”*

In the second phase, social outcomes remained similar. Participants continued speaking about how the strength of TBI-Health was related to the inclusive nature of the program for people with all ranges of abilities, in addition exchanging socially with members outside of the program. M2 shared his perception about, “establishing a contact with them (other YMCA members), many of us even made some good friends, chatting with people who don’t belong to our group, I’ve noticed …” Then, in phase three, the participants discussed how the program brought them together in solidarity. P2 explained, “We help each other a lot. Because, with TBI … you become, how can I say, a little less confident maybe … So being together makes you feel a little more solid.” By this final phase, participants were discussing increased confidence to expand their social circle through their program participation and the outdoor 5k sport challenge. P5 spoke about a member who started assisting her by running arm-in-arm on the YMCA track, “as for me … I found someone to help me out every session, and he’s almost always there … he’s a member of the YMCA but from another group … we’d like to do something outside of the gym, but it hasn’t happened yet. This way I have a friend though, that I can meet outside.”

### Physical Activity Autonomy – “I realized that I have more autonomy in my training, I know more where I am going. So, I can do my things and I can adapt them” (P7)

The ***PA Autonomy*** theme highlighted the perceived transitions that occurred in knowledge, abilities, and sex- and gender-related beliefs relating to exercise and sport participation within and outside of the gym, which provided the participants with the confidence and independence to be more autonomous in their PA participation. Participants discussed the evolution in their sex- and gender-related beliefs and confidence about PA participation, as captured in the *Sex and Gender* sub-theme. For instance, in phase one, preconceived sex- and gender-related beliefs about preferences for type and intensity of PA were identified. Participants commented about their sex- or gender-based observations. M1, a female mentor, remarked that “I think some of the men are always working hard training, and women sometimes just do more social.” Others discussed sex- or gender-based observations about exercise preferences. P12 added, *“*it seems normal to me that guys prefer certain exercises, but not women. Even in (fitness) classes, it’s like that.”

Interestingly, in phase two, participants began challenging their own preconceived sex- and gender-related beliefs through a newfound appreciation for different PA modalities, incentives, and preferences. M3 asserted, “there’s no gender! [Everyone laughing]. Gender wise! I mean, there’s like something here that brings the group together. And my preference is to come here to the YMCA to see you guys.” However, M1, the mentor, still felt that “they (female members) just like to always say like they don’t want to do something because they’re busy talking. They’re not even exercising.” And while P12 first remarked that “I was always under the impression that guys were more about strength training, and girls it’s more cardio.” she further added, “finally discovered that I loved weight training. It’s fun! I neglected that … but no, it sure helps because I no longer need orthotics.”

By phase three, the participants did not speak about as many differences and continued to challenge what they perceived to be others’ preconceived sex- and gender-related beliefs about personal exercise preferences in the gym. Apparently, the groups’ sex- and gender-related beliefs had changed. For instance, P11 concluded that “if you look carefully at the clientele up there, you’ll see that there are many women, and women who train. Some are even body building. It’s crazy … you’ll see all sorts” P12 shared a personal example,

Exercises like barbell lifting, all those exercises I had, there were just guys doing them. And the guys, it seemed, were staring at me … (imagining them saying) “What do you think you’re doing? You shouldn’t be here.” - I'm doing my workout, that's all. So basically, you could say there is a zone for women and a zone for men (in the gym), but for me, I like to be able to do all the exercises …

P3 further added, “girls … guys, it’s all the same,” thereby demonstrating, once again, how their previous impressions about the sex- and gender-related beliefs about exercise had changed.

Within the ***PA Autonomy*** theme, participants also discussed their capability as a gym-goer and knowledge of PA benefits after a TBI that supported independent PA, as highlighted in the *PA Capability* sub-theme. P8 shared how following and remembering a PA program, “it makes you realize you can do it.” Then P13 added, “for sure, differently, but nevertheless, you can do it.” Participants felt that others should be informed about the PA benefits after TBI too. P12 explained, “when you’ve been in the hospital for a few months because you’re not able to function, they (doctors) should be the ones to tell us: you should be moving. It will help you with your concentration, it will help you.” Then, in phase two, participants noted how the self-discipline of coming to the gym twice a week, as well as their increased PA knowledge, confidence, autonomy, and freedom contributed to their overall ability to participate in PA. P2 explained, “I think we all realized that we were able to do our activities on our own, well it gives you, it gives confidence.” Participants also discussed how their newfound confidence made them curious to try out new forms of PA, in different places, like the larger gym on the third floor or the walking trail. For example, P6 shared: “in phase two, I find there is greater freedom, you train all by yourself, you look for the machines you’re more …” P11 added “at ease with,” and P6 continued: “more successful with.”

In phase three, participants began identifying the increased capability and freedom associated with their independent exercise, in addition to an appreciation for outdoor PA, and a desire to explore new PA avenues (such as creating a sports team), as captured in the *PA Capability* sub-theme. Notably, they discussed how they began enjoying the run/walk activities in preparation for the 5k sport challenge. M3 shared, “It’s a good idea to integrate that (outdoor outings) in the program … I think it’s super important to get fresh air outside … makes your worries go away, as they say.” The participants also discussed how their increased autonomy and freedom allowed them to experience more pleasure from doing PA. P12 explained, “now we’re a lot more familiar with the YMCA and we all know that we’re good at doing what we’ve learnt to do. So, it’s just more exciting, and we’re just able to go and have fun.” There was even an interest to start something of their own, “I was asking myself if it is possible, next year for example, to have a group within our group, like in a special team, like basketball for example, and they play against another group that has difficulties too” (M2). Based on their examples, it was evident participants wanted to continue their PA participation in and outside the program.

### Results Integrated within the final program

Suggested modifications from the focus groups were incorporated in the final version of the program. For instance, the existing program schedule, reminders, and supports (e.g., whiteboards, mobile applications) were maintained. However, newly integrated items in the program manual included providing French and English versions, clearly outlining the objectives and step-by-step progressions within each program phase, as well as defining the mentors’ and participants’ roles and responsibilities with a code of conduct. To further promote participation, the mentors began visiting group classes provided at the YMCA (e.g., yoga, spinning) with participants outside program hours, and the run/walk outings in preparation for the sport challenge were divided into walking speed groups depending on the participants’ abilities. To advertise the program externally, TBI-Health was showcased at provincial and national conferences, with support from the mentors as spokespersons.

## Discussion

The purpose of this interpretive case study was to provide an in-depth account of the experiences and outcomes of the participation in and co-creation of a new community-based TBI-Health program to enhance sport and exercise participation for adults with moderate-to-severe TBI. The results identified themes, which will help enhance the program, and can inform future research and practice that promotes participation after moderate-to-severe TBI.

The program participants discussed feeling anxious about participating correctly in PA, by comparing themselves physically to other members in the gym-based environment and commenting about invisible difficulties with fatigue after TBI. This finding is relevant because participation and long-term recovery after TBI may be influenced by social comparisons with peers, caregivers, and others after injury. For example, comparing oneself to an individual who is functioning well (upward comparison) can be positive if it is inspiring, but it can also be negative if the situation highlights one's deficits (23). The latter example may therefore present a barrier to participating in community-based activities for health benefits after TBI if the upward comparison is associated with unwanted feelings. The social comparisons in our study, consistent with the concept of upward comparisons, are in line with similar findings about community-based PA after severe TBI (13), which indicates a reoccurring PA barrier.

Ponsford and colleagues (24) suggest that self-concept and self-esteem are negatively influenced after brain injury and propose a clinical focus on specific self-concept dimensions to improve self-esteem. However, the program barriers associated with the initial anxiety about participating properly in PA, upward comparisons, and TBI-related difficulties with fatigue were largely reduced by phase three of our program, which suggests that social comparisons may also diminish through participation in peer-assisted community programs. The peer component is central to the TBI-Health program and future research could examine how similar peer-assisted PA programs can support self-concept and self-esteem after moderate-to-severe TBI as a cost-efficient method of improving participation in the community.

Study participants emphasized the types of external facilitators that enabled their program participation, such as the structured framework in place that afforded support and guidance from the program peer mentors, YMCA staff, and research team. In this way, having structure and routines to follow facilitated their involvement in PA. An appreciation for structure may be linked to the types of sequelae with which people with TBI live, including difficulties with social cognition and dysexecutive behavior, which are both shown to be significant predictors of vocational and social participation after moderate-to-severe TBI (25).

Program aids, like clear instructions and visual reminders were emphasized by participants to facilitate their involvement and execution of program exercises. Having access to technology aids resonated with participants as an extra tool to help them understand how to carry out exercises and what muscles were being used in the process. These findings are important for PA participation after moderate-to-severe TBI in the long-term/chronic period because frequently reported problems include memory difficulties, and problems related to cognitive aspects of executive functioning (26). Thus, incorporating simple, yet specific program aids to better understand and remember exercises could be an effective strategy for promoting and extending PA participation after moderate-to-severe TBI in other community-based contexts.

Motivation after TBI is a multi-dimensional problem because studies have consistently shown that injuries to the brain commonly lead to apathy, or a decrease in the cognitive and psychological processes involved with goal-directed activity (27). However, a deeper understanding about the inner mechanisms that distinguish whether neural or psychological constructs are responsible is less known (28). Similarly, a lack of motivation was cited as one of the most frequently reported PA barriers (≥42%) for adults with moderate-to-severe TBI aged 18–55 years and older (29).

Interestingly, our participants' motivation to participate in the adapted PA program evolved over the course of the intervention, as revealed by the similarities and differences across the three time points. The support and accountability afforded by the group-based activities stood out, because participants discussed overcoming motivational difficulties thanks to the positive influence of training with peers. This suggests that motivational problems rooted in psychological mechanisms may be attenuated through peer-assisted activities. Similar outcomes about the ways in which community-based peer-support programs can have a positive impact on recovery after TBI through acceptance and encouragement have been identified (30). Our findings extend those results with the positive influence that peers can have on motivation to participate in health-related PA behaviors in the community after moderate-to-severe TBI.

In the community-based context, tailoring a PA-based health promotion program for adults with moderate-to-severe TBI through stakeholder input is relevant to its overall success (11). Based on the program modifications discussed and the participants' active engagement throughout the 9-month intervention, our findings suggest that a structured, progressive, and tailored approach to promoting PA participation in community for adults with moderate-to-severe TBI is favorable and should be maintained within the TBI-Health program. However, similar peer-assisted programs in the community context have demonstrated potential for social reintegration after moderate-to-severe TBI in a shorter six-month period (31). Therefore, it may be relevant to examine whether the TBI-Health program with a shorter duration (and thus perhaps less costly) may provide similar benefits with participation.

Our participants discussed perceived improvements with their cognitive functions beginning in the second phase of the program, with examples about how training programs engaged their concentration and memory. Studies that investigate the potential impact of cardiovascular exercise on memory consistently show promise (32), although two important factors need to be addressed: the timing and type of exercise involved, which relates to a single exercise stimulus vs. multiple exposures (33). The improvements with memory discussed by our participants after six months of being involved in the program (end of phase 2) would therefore suggest these kinds of changes may be consistent with repeated exposure through long-term exercise participation. It is also noteworthy that resistance-training exercise programs were central to the cognitive improvements perceived by participants. Resistance-training exercise may indeed provide cognitive benefits (34, 35) and although some studies have examined resistance training after TBI (36, 37), there is a general lack of focus on the impact this may have with cognitive function, including memory. Therefore, based on the comments about improved memory beginning in the second phase, and related to their resistance training programs, this underscores the importance of continued PA participation several years after TBI.

Based on the findings about the perceived social outcomes, it was apparent that participants felt comfortable in their social environment. This is important because community-based programs have been proposed as the logical transition from the rehabilitation continuum for people living with disabilities (38) and few studies have examined the specific effect that an individual's environment (e.g., social network members) can have on activity resumption (39). Our participants further discussed “inclusivity” and “solidarity” in the second and third phases, in addition to making good friends and planning outside activities with other members of the YMCA. These findings are consistent with expert social worker perspectives about the importance of “inclusion” and “activity enablement” for the influential role that social networks play in activity resumption after brain injury (40), but more importantly, is the way in which these relationships were developed organically, and as the direct result of PA participation in a community-based YMCA fitness centre. Based on the perceived experiences of participants, YMCA fitness centres may provide the necessary environment to promote social participation after moderate-to-severe TBI, which is central to the success of our program. Future research should examine how YMCA fitness centres can promote social reintegration as an extension to the rehabilitation continuum of care.

The transitions in sex- and gender-related beliefs about PA participation are noteworthy because sex- and gender-based stereotypes may present barriers to recommended amounts of PA (e.g., 150 min of moderate aerobic exercise + muscle strengthening activities ≥2 days a week; 41, 42). In a similar context, a systematic review and meta-analysis that investigated the presence of gender differences in PA for adults with intellectual disabilities demonstrated that men engage in more PA, which is also reflective of the general population (43). Therefore, it is plausible that similar PA differences exist for adults who live with TBI. Our participants' initial perceptions about gender-related assumptions with PA intensity and type further support this theory, emphasized by the perceived transitions in sex- and gender-related beliefs in phase two and three of our program. These findings further highlights the possibility of equitable sex and gender participation in various forms of health-related PA after moderate-to-severe TBI, a research gap identified in community-based PA programs (44).

There is a recognized need to develop interventions that assist with the maintenance of health-related PA behaviors after brain injury (45). Within the PA Autonomy theme, reoccurring sub-themes related to feeling capable and autonomous were associated with the participants’ enhanced pleasure and interest in group-based PA. These findings highlight factors that may be associated with autonomy-supported behavior, a central tenet of self-determination theory (SDT), which suggests that autonomy-motivated behavior is supported through the satisfaction of basic psychological needs for autonomy, competence, and relatedness (46). Although, SDT is less recognized for a comprehensive understanding of motivation after TBI, our participants' examples related to the capability and freedom associated with their group-based PA clearly led to an interest to explore new ways of exercising in and outside of the gym. Therefore, autonomy-supportive behaviours may be recognized as a central component of the TBI-Health program, which future research may examine for the promotion and maintenance of PA participation in the community-based context after moderate-to-severe TBI.

## Limitations

The results may be limited to the individuals involved with the community-based TBI-Health program, which may not be representative of other groups of adults with moderate-to-severe TBI or other community-based fitness centres. However, a unique strength of this study was prolonged engagement with the study participants, which provided a rich and in-depth account of how their perceptions transitioned over 9-months of the participatory co-creation process. Another limitation of this study is related to positivity bias – the possible tendency for study participants to report positive views, expectations, and information about the community-based program, whether related to their own involvement, their relationship to the PI who led the program, or a general desire to please others involved. However, with the assistance of an analyst with no prior relationship with participants, and critical friends on the research team, we attempted to provide a transparent account of all our methods to ensure the data, representations, and outcome accurately reflected the participants' experiences. Finally, although prepared with the consolidated criteria for reporting qualitative studies (COREQ) guidelines, the reflexive thematic analysis undertaken here fully embraces the subjective skills researchers bring to the interpretive process. The open coding and inductive theme development did not seek to ensure the accuracy and reliability of the process through coder agreement, but rather through a reflexive, critical, and iterative process informed by the team of authors. Therefore, codes and themes may be different for other analysts.

## Conclusions

This study identified three themes across the participatory co-creation experience of a 9-month 3-phased adapted PA program for adults with moderate-to-severe TBI, which highlighted the participants' Program Participation, Biopsychosocial Changes, and PA Autonomy. Findings from this study were integral to the design and implementation of the new and enhanced TBI-Health program and provide evidence-based recommendations for promoting continued PA participation after neurotrauma. We hope that future research and practice related to the design and implementation of community-based PA interventions will undertake similar methodological approaches to partnering with key stakeholders, based on the associated benefits with mood, quality of life and participation that may be obtained throughout the collaborative process.

## Data Availability

The raw data supporting the conclusions of this article will be made available by the authors, without undue reservation.
