# Supplementary material for: Participatory co-creation of an adapted physical activity program for adults with moderate-to-severe traumatic brain injury
Source: Front Rehabil Sci. 2022 Aug 4;3:900178. doi: 10.3389/fresc.2022.900178 (PMC9397937; doi:10.3389/fresc.2022.900178)
Supplement: Supplementary file 1 [file Image_1_v1.pdf]

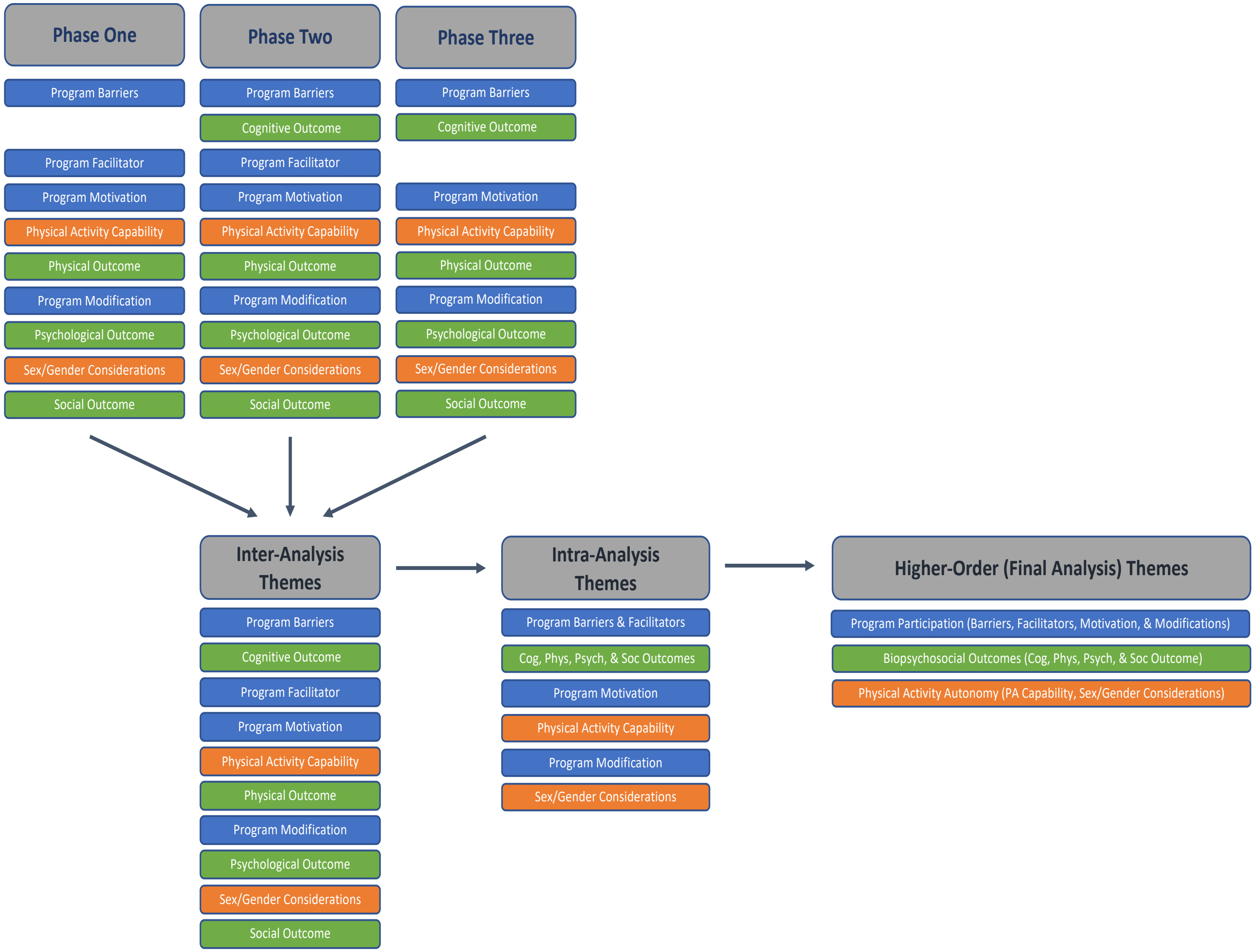

Supplemental File 2: Graphic Report of Intra- and Inter-Analysis Themes
